# Supplementary material for: The Protection and Management of Wapiti in Desert Oases: Bare Land Poses a Limitation to Wapiti Conservation
Source: Biology (Basel). 2024 Sep 20;13(9):737. doi: 10.3390/biology13090737 (PMC11428931; doi:10.3390/biology13090737)
Supplement: Supplementary file 1 [file biology-13-00737-s001.zip › biology-3199636-supplementary.pdf]

Table S1. Habitat selection factors for Alashan wapiti.

Class	Variable	Abb	Reason	Source
Landcover	Crop	Crop	Land use practices are one of the primary factors affecting wildlife habitats. [1]。	（https://data.casearth.cn/）
	Deciduous broad leaved forest	DBF		
	Evergreen needle leaved forest	ENF		
	Shrubland	Shrubland		
	Grass land	Grassland		
	Wetland	Wetland		
	Impervious surfaces	Imper_surface		
	Bare area	Barearea		
	Water body	Waterbody		
Topographic	Elevation	ELE	Topography plays a crucial role in wildlife habitat selection, and variations in terrain are often closely correlated with vegetation patterns and anthropogenic disturbances. [2]。	http://dwtkns.com/srtm30m
	Compound Topographic Index	CTI		
	Aspsct	Aspsct		
	Slope	Slope		
	Terrain Ruggedness Index	TRI		
Human activity	Distance to Highways	DisHighways	Anthropogenic disturbances are the primary cause of habitat fragmentation, which severely impedes species migration and dispersal. [3,4]。	https://www.webmap.cn/
	Distance to Primary road	DisPrimaryroad		
	Distance to Other road	DisOtherroad		
	Distance to Village	DisVillage		

Class	Variable	Abb	Reason	Source
Food resources	Normalized Difference Vegetation Index	NDVIsummer	Vegetation productivity is an indicator of food availability, which is a critical factor in habitat suitability and is positively correlated with changes in animal populations. [5]。	https://modis.gsfc.nasa.gov/
		NDVIwinter		
		NDVIwintermin		
Climate factor	Annual Mean Temperature	Bio_1	Forage quality affects physiological condition, population dynamics, habitat use, and distribution of ungulates. Precipitation and temperature are associated with forage growth and abundance, and are also associated with red deer reproduction[6].	https://worldclim.org/
	Temperature Seasonality	Bio_4		
	Temperature Annual Range	Bio_7		
	Mean Temperature of Driest Quarter	Bio_9		
	Mean Temperature of Coldest Quarter	Bio_11		
	Annual Precipitation	Bio_12		
	Precipitation Seasonality	Bio_15		
	Precipitation of Coldest Quarter	Bio_19		

1. Cimatti, M.; Ranc, N.; Benítez-López, A.; Maiorano, L.; Boitani, L.; Cagnacci, F.; Čengić, M.; Ciucci, P.; Huijbregts, M.A.; Krofel, M. Large Carnivore Expansion in Europe Is Associated with Human Population Density and Land Cover Changes. *Divers. Distrib.* **2021**, *27*, 602–617. doi:10.1111/ddi.13219
2. Suel, H. Brown Bear (*Ursus Arctos*) Habitat Suitability Modelling and Mapping. *Appl. Ecol. Environ. Res.* **2019**, *17*, 4245–4255.doi: 10.15666/aeer/1702_42454255
3. Parsons, B.M.; Wilson, A.E.; Graham, K.; Stenhouse, G.B. Grizzly Bear (*Ursus Arctos*) Movements and Habitat Use Predict Human-Caused Mortality across Temporal Scales. *Can. J. Zool.* **2022**. doi: 10.1139/cjz-2022-0054
4. Hubbard, T.; Cove, M.V.; Lafferty, D.J. Human Recreation Impacts Seasonal Activity and Occupancy of American Black Bears (*Ursus Americanus*) across the Anthropogenic-Wildland Interface. *Sci. Rep.* **2022**, *12*, 1–11. doi:10.1038/s41598-022-15665-x
5. Hoeks, S.; Huijbregts, M.A.; Busana, M.; Harfoot, M.B.; Svenning, J.-C.; Santini, L. Mechanistic Insights into the Role of Large Carnivores for Ecosystem Structure and Functioning. *Ecography* **2020**, *43*, 1752–1763. doi: 10.1111/ecog.05191
6. Marshal, J.P.; Krausman, P.R.; Bleich, V.C. Rainfall, Temperature, and Forage Dynamics Affect Nutritional Quality of Desert Mule Deer Forage. *Rangel. Ecol. Manag.* **2005**, *58*, 360–365, doi:10.2111/1551-5028(2005)058[0360:RTAFDA]2.0.CO;2.
